# *Pseudomonas aeruginosa* Pathogenicity and Its Interaction with Other Microorganisms During the Skin Wound Healing Process

**DOI:** 10.3390/ijms26199677

**Published:** 2025-10-04

**Authors:** Inti Yamberla, Carla Pupiales, Andrea Jazmín Chiliquinga, Tania Sulca-Villamarín, Alejandra Plasencia, Francisco Cabrera Aulestia, Ramiro F. Díaz, Andrés Caicedo, Pedro Miguel Barba

**Affiliations:** 1 Carrera de Biotecnología, Universidad Técnica del Norte, Ibarra 100105, Ecuador; yuyamber@gmail.com (I.Y.); carlamirecp@gmail.com (C.P.); ajchiliquinga1@utn.edu.ec (A.J.C.); tssulca@utn.edu.ec (T.S.-V.); ale.kikyo28@gmail.com (A.P.); 2 Instituto de Investigaciones en Biomedicina iBiomed, Universidad San Francisco de Quito, Quito 170901, Ecuador; fcabrera@usfq.edu.ec (F.C.A.); rdiaz@usfq.edu.ec (R.F.D.); acaicedo@usfq.edu.ec (A.C.); 3 Mito-Act Research Consortium, Quito 170901, Ecuador; 4 Laboratorio de Salud Animal, Instituto de Biodiversidad Tropical, Escuela de Medicina Veterinaria, Universidad San Francisco de Quito, Quito 170901, Ecuador; 5 Escuela de Medicina Veterinaria, Universidad San Francisco de Quito, Quito 170901, Ecuador; 6 Escuela de Medicina, Colegio de Ciencias de la Salud, Universidad San Francisco de Quito, Quito 170901, Ecuador; 7 USFQ Space Front, Universidad San Francisco de Quito, Quito 170901, Ecuador

**Keywords:** *Pseudomonas aeruginosa*, skin wound healing, pathogenesis, biofilm, immune system

## Abstract

*Pseudomonas aeruginosa* is a Gram-negative opportunistic pathogen frequently associated with delayed wound healing, particularly in chronic skin injuries. Its capability to form biofilms, secrete virulence factors, and the faculty to compete with other microorganisms makes it a major challenge in clinical wound management. Recent literature reveals different molecular and cellular mechanisms through which *P. aeruginosa* disrupts the wound healing process. Findings highlight that it interferes with key phases of healing by modulating host immune responses, degrading extracellular matrix components, and inhibiting keratinocyte migration. Its quorum-sensing systems regulate the expression of critical virulence factors such as exotoxin A, elastases, pyocyanin, and rhamnolipids. Additionally, the production of the biofilm matrix components alginate, and polysaccharides provide protection against host defenses and antibiotics. Interactions with other microorganisms, including antagonistic effects on *Staphylococcus epidermidis* and synergistic relationships with *Staphylococcus aureus*, modify the wound microbiota. Promising therapeutic alternatives have shown efficacy in disrupting biofilms and reducing virulence. These insights remark the importance of targeting both *P. aeruginosa* and its ecological interactions to enhance wound healing outcomes and develop more effective treatments. This review aimed to highlight the pathogenic role of *P. aeruginosa* and its interactions with other microbial species in the context of skin wound healing.

## 1. Introduction

Wound healing is a complex and dynamic biological process involving a coordinated cascade of cellular and molecular events that restore the integrity of damaged skin. This process occurs in overlapping stages, including hemostasis, inflammation, proliferation, and remodeling, all of which depend on a tightly regulated environment and the interplay of local and systemic factors [1,2].

When skin integrity is compromised, the balance of the local microbiota is disrupted, making the wound more susceptible to colonization by opportunistic and pathogenic microorganisms [3,4]. Among these pathogens, *Pseudomonas aeruginosa* is particularly notable for its ability to colonize open wounds and interfere with the wound healing trajectory. Its presence not only alters the local microbial community but also interferes with the progression of healing by influencing immune responses and delaying resolution of inflammation [5,6]. The microbial shift caused by *P. aeruginosa* colonization often leads to competitive interactions with other microorganisms such as *Staphylococcus aureus*, *Klebsiella pneumoniae*, and *Streptococcus pyogenes*, further complicating infection outcomes [7,8].

The wound environmental conditions particularly elevated iron concentrations, contributing significantly to the pathogenic potential of *P. aeruginosa*. High iron levels in the wound microenvironment have been shown to inhibit the repressor proteins of Psl (Polysaccharide of the biofilm matrix) biosynthesis, molecule crucial for biofilm development, and bacterial aggregation [9]. Moreover, iron availability itself plays a fundamental role in improving the ability of the pathogen to evade host immune responses and persist in chronic wound settings [10,11].

Understanding how *P. aeruginosa* interacts with both host tissues and co-infecting microorganisms is essential for developing new therapeutic approaches. Despite numerous studies on its virulence, there is still limited integrative knowledge regarding how *P. aeruginosa* dynamically interacts with other microbes within the wound niche and how this affects healing outcomes. Therefore, this study aims to investigate the pathogenic role of *P. aeruginosa* and its interactions with other microorganisms in skin wound healing.

## 2. Brief Overview of the Normal Skin Wound Healing Process

Wound healing is a natural physiological process that takes place to repair damaged tissues and involves multiple molecular, cellular, and clinical facts. This process includes hemostasis, inflammation, proliferation, and remodeling phases [1]. Wound healing begins immediately after tissue injury with the activation of hemostasis, which results in the formation of a fibrin mesh, a protein-based scaffold that halts bleeding and provides a structural matrix for cell adhesion and signal integration. This fibrin network also facilitates the release and concentration of chemical mediators known as growth factors, which are signaling proteins that regulate key cellular behaviors such as proliferation, migration, and differentiation [12,13].

The inflammatory phase follows the hemostasis stage and is characterized by the infiltration of neutrophils and macrophages into the wounded area [14,15]. These immune cells initially try to eliminate the pathogens by phagocytosis, also the neutrophils build an extracellular matrix to capture opportunistic pathogens such as *Pseudomonas aeruginosa* [16]. On the other hand, the macrophages have more versatile actions, such as contributing to the secretion of pro-inflammatory cytokines such as IL-1, IL-1β, IL-6, and TNF (Tumor Necrosis Factor); macrophages also recruit other leukocytes to the wound area and prepare the zone for tissue regeneration by TGF (Transforming Growth Factor) excretion [17].

During the proliferative phase, keratinocytes, fibroblasts, and myofibroblasts migrate and proliferate to restore epithelial integrity and synthesize extracellular matrix components essential for tissue repair [18,19].

In the remodeling phase, the granulation tissue is reorganized and replaced by a more structured extracellular matrix, leading to revascularization, reduction in cellularity, and partial restoration of tissue function. Although these stages occur in a sequential order, they are not entirely distinct and may overlap temporally depending on the nature and severity of the injury [20]. Proper resolution of each phase is critical to achieving functional tissue regeneration and preventing chronic wound formation.

The pathogenic potential of *P. aeruginosa* becomes particularly relevant during the inflammatory and proliferative phases by secreting exotoxins and forming biofilms, *P. aeruginosa* can disrupt immune responses, prolong inflammation, and hinder keratinocyte migration, ultimately delaying wound closure and promoting chronic infection [21,22].

## 3. The *Pseudomonas aeruginosa* Role in Skin Wound Healing

*Pseudomonas aeruginosa* is a Gram-negative opportunistic pathogen capable of adapting to a wide range of environmental and host-associated niches. It possesses a broad arsenal of virulence mechanisms that enable colonization, immune evasion, and tissue destruction.

One of the key adaptive traits of *P. aeruginosa* is the ability to interact with host defense systems, particularly by binding to vitronectin glycoprotein. Normally, vitronectin is located on the extracellular matrix (ECM) and regulates the complement immune pathway and the recruitment of defense cells. This interaction between the bacteria with the vitronectin affects the immune cell adhesion to the damage zone and facilitates the immune response evasion and contributes to bacterial adhesion to host tissues [23,24].

In acute and chronic wounds, *P. aeruginosa* thrives by producing a complex biofilm structure constituted by polysaccharides such as alginate, Pel, and Psl (Table 1). These biofilms are further stabilized by extracellular DNA, structural proteins, fimbriae and type IV pili protein, this context facilitates surface adherence and microcolony formation by the pathogen [25,26]. The biofilm not only enhances antibiotic resistance but also physically shields bacteria from immune cells such as neutrophils and macrophages, and finally interferes with complement pathway, exacerbating chronic inflammation [7,27].

**Figure 1:**
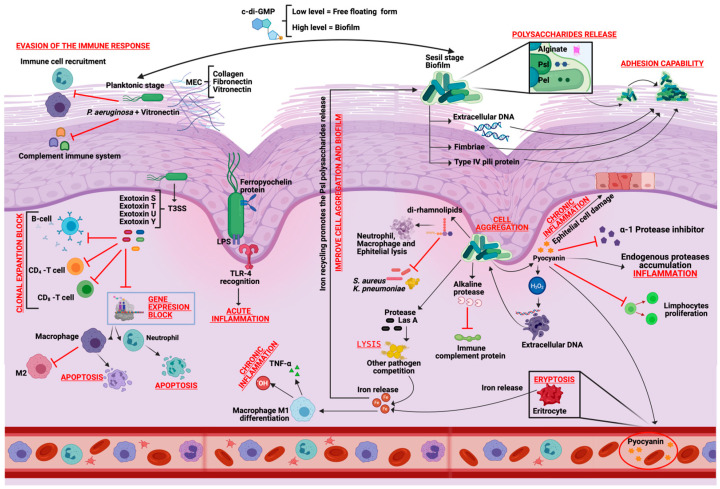
Pathogenicity of *Pseudomonas aeruginosa* in wounds healing process.

*P. aeruginosa* also secretes an array of extracellular proteins and toxins that intensify its virulence. Among them are pyocyanin, elastase, and alkaline protease, all of which contribute to oxidative stress, tissue degradation, and modulation of host immune signaling [28]. A pivotal contributor to pathogenicity is the Type III Secretion System (T3SS), a needle-like apparatus that injects effector proteins directly into host cells. T3SS system delivers specific exotoxins such as ExoS, ExoT, ExoU, and ExoY, each of which disrupts distinct cellular processes like cytoskeletal integrity, intracellular signaling, and apoptosis (Figure 1) [6,29].

Additional virulence factors include phospholipase C, which lyses host cell membranes, the ferripyochelin-binding protein involved in iron acquisition, and the surface-expressed lipopolysaccharide (LPS), which stimulates strong inflammatory responses through Toll-like receptor 4 activation [30]. These components together enable *P. aeruginosa* to manipulate both the immune landscape and the structural integrity of wounded tissue, thereby delaying resolution and contributing to chronic wound pathology (Table 1).

**Table 1:** Main molecules secreted by *Pseudomonas aeruginosa* during the wound healing process.

Cells	Secreted Molecules	Description	Reference
*Pseudomonas aeruginosa*			
Planktonic cell	Exotoxin A	Exotoxin that inhibits protein synthesis and causes cytopathic effects in immune cells.	[31]
Sessile cells (Biofilm)	Pyocyanin	Virulence factor that induces eryptosis. It has a potential role in biofilm formation by promoting eDNA release due to cell lysis.	[32]
	Alkaline protease	Extracellular protease, that prevents bacterial elimination by degrading the immune C2 complement protein, can also degrade flagellin and is a known pro-inflammatory responses activator.	[33]
	Di-rhamnolipid	Glycolipid biosurfactant that lyse neutrophils, macrophages, and different animal cells rapidly. Acts in swarming motility and shape the biofilm structure, also possess different antimicrobial activity.	[32]
	Cyclic diguanosine-5′-monophosphate (c-di-GMP)	Nucleotide on which the lifestyle of *P. aeruginosa* depends; low levels favor the motility factors expression, promoting the planktonic state and high levels favor the sessile lifestyle by increasing the extracellular matrix components and adhesion factors expression.	[32]
	Alginate	Mannuronic acid and glucuronic acid linear polymer, a biofilm component and acts as a cell evasion mechanism, blocking the antibodies and phagocytosis immune action.	[34]
	Lectin B	Membrane protein that coats the bacterial cells together and promotes the adhesion of *P. aeruginosa* to both host cell and exopolysaccharide matrix. On epithelial cells, it inhibits cell migration and proliferation during re-epithelization phase.	[35]
	Quinolones	Antimicrobial molecules with selective toxicity to inhibit the synthesis of other bacterial DNA.	[36]
	Pel	Polysaccharide involved binding initiation on the surface and maintenance the integrity of biofilm. It crosslinks the eDNA in the biofilm matrix and maintains cell–cell interactions.	[25]
	Psl	Polysaccharide involved in cell–cell adhesion. It reduces the immune system attacks because it inhibits opsonization and reduces the neutrophil’s reactive oxygen species (ROS). In addition, it reduces the matrix phagocytosis.	[25]
	Acetate	Molecules produced by *Pseudomonas aeruginosa*, useful to bind to the LPS side chains or alginate by ester bond for preventing the complement immune system activation.	[34]

## 4. Pathogenicity of *Pseudomonas aeruginosa* in Skin Wound Healing

The pathogenicity of *Pseudomonas aeruginosa* in cutaneous wounds is supported by a diverse set of molecular and physiological strategies that enhance its ability to persist, evade host defenses, and disrupt the healing environment. One important factor is the presence of outer membrane porins, particularly the OprF porin, which functions as a nonspecific diffusion channel involved in nutrient acquisition and environmental sensing [37]. The OprF porin has also been linked to the regulation and export of virulence molecules such as pyocyanin, a redox-active metabolite that generates oxidative stress in host cells and inhibits the growth of competing microorganisms [38].

Quorum sensing (QS) is a central mechanism in *P. aeruginosa* pathogenesis that modulates the expression of genes associated with virulence and biofilm maturation, it is a cell-density-dependent regulatory system. *P. aeruginosa* possesses at least two canonical QS circuits based on acyl homoserine lactones: the “las” and “rhl” systems, composed of the *lasI*/*lasR* and *rhlI*/*rhlR* gene pairs, respectively [39,40]. These QS pathways activate the expression and release of numerous virulence factors, including *toxA* (exotoxin A), *aprA* (alkaline protease), and *lasA* and *lasB* (elastases), which contribute to tissue destruction and immune interference [41]. The rhl system also regulates motility and biosurfactant production by activating the *rhlAB* operon, which controls the expression and synthesis of molecules as di-rhamnolipids that reduce surface tension in the tissues and facilitate bacterial spreading [35,42].

Another key regulatory mechanism is the intracellular signaling molecule cyclic diguanosine monophosphate (c-di-GMP), which modulates the transition between planktonic and biofilm lifestyles of *P. aeruginosa*. High intracellular concentrations of c-di-GMP promote sessile behavior by enhancing the production of extracellular polymeric substances, including alginate, Pel, and Psl polysaccharides, and by inducing cell aggregation and adhesion to surfaces [43]. Conversely, low levels of c-di-GMP increase motility and reduce biofilm formation, facilitating dispersal and new colonization [44]. This regulatory system plays a pivotal role in modulating virulence in response to environmental cues within the wound (Figure 1) [45,46].

The expression of these virulence factors enables *P. aeruginosa* not only to resist phagocytic clearance and the immune antimicrobial activity but also to manipulate the wound microenvironment. This includes disruption of immune signaling, degradation of extracellular matrix components, and modulation of host cell migration and proliferation. As such, understanding these interlinked regulatory networks provides a baseline for designing targeted therapies directed to disrupt quorum sensing, inhibiting biofilm formation, or restoring immune balance in infected wounds [6,8].

## 5. *Pseudomonas aeruginosa* Biofilm Effect on Skin Wounds

The *P. aeruginosa* biofilm formation is an aggravating key factor for the inflammatory response and impairing skin wound healing [5,35,47]. The biofilm produced by *P. aeruginosa* promotes the bacterial persistence ability, facilitating the bacterial wound colonization [39,47]. *P. aeruginosa* biofilms are generally constituted by exopolysaccharides like alginate, Psl and Pel polysaccharides, exogenous DNA, and lipids, which determine the biofilm stability and protect the bacteria from the host immune response [48,49].

On the other hand, *P. aeruginosa* mature biofilms often form a central core surrounded by a distinct shell-like wall of non-motile cells [50]. In the biofilm, there are two different bacterial phenotypes, highly motile cells in the central core, and planktonic non-motile cells on shell-like wall [51]. At the same time *P. aeruginosa* produce alginate that confers bacterial resistance by lipopolysaccharide (LPS) side chains elimination to obtain acetate substitutes [34,52]. The acetate residues are attached via ester bonds to hydroxyl groups that act as covalent bond acceptors for opsonins on the bacteria surface, preventing the activation of the immune complement system.

These biofilms adhere to devitalized tissue and wound bed surfaces, often forming stratified layers in which metabolically active bacteria reside in the outer regions while dormant or persistent cells occupy the deeper layers, enhancing antibiotic tolerance [50,51]. In the wound microenvironment, the biofilm matrix not only provides a physical barrier to immune cell penetration but also retains pro-inflammatory mediators, perpetuating tissue damage and delaying re-epithelialization [52]. This structural adaptation to the skin wound milieu differs from flow-cell or in vitro models, as wound biofilms interact closely with host extracellular matrix components and inflammatory exudates, which influence their architecture and stability [25,50].

Furthermore, the Pel and Psl polysaccharides are essential for subpopulation interactions and microcolony formation in the later stages of biofilm formation. Pel inhibits opsonization, resulting in reduced production of neutrophil reactive oxygen species (ROS) and decreased matrix destruction by phagocytes cells [25].

The release of Lectin B is another important virulence factor that causes loss of epithelial polarity. It has a high binding affinity for L-fucose and its derivatives [47]. The process that LecB binds to fucose-bearing lipids induces membrane invaginations and locates integrin’s in these invaginations. As consequence, LecB causes the inhibition of cell migration, facilitates the establishment and stabilization of bacterial infection, and prevents proper healing [53].

The LecB not only induces membrane invaginations that trap integrins but also disrupts essential signaling pathways involved in wound healing. By prolonging the inflammatory phase, it impairs neutrophil and macrophage transition to pro-repair phenotypes, which delays angiogenesis and tissue regeneration. Additionally, LecB contributes to excessive NET formation and inflammasome activation in macrophages, further exacerbating inflammation and preventing resolution, especially in chronic wounds such as diabetic ulcers [16].

## 6. *Pseudomonas aeruginosa* Interaction with the Immune System

A successful bacterial infection develops when the immune response fails. Also, it is well known that persistent inflammation in wounds is accompanied by an inefficient healing process [35,54]. *P. aeruginosa* may alter the inflammatory response by secreting pro-apoptotic factors, which are correlated with an unresolved inflammation [55]. For example, exotoxin A modifies the gene expression in mammalian cells and causes polymorphonuclear neutrophils (PMNs) and macrophages apoptosis [56]. When PMNs die, the cellular components are released, increasing inflammation and causing host collateral damage. Similarly, in macrophages absence, the fragmented neutrophils will eventually release their pro-inflammatory content [57] (Table 2).

**Figure 2:**
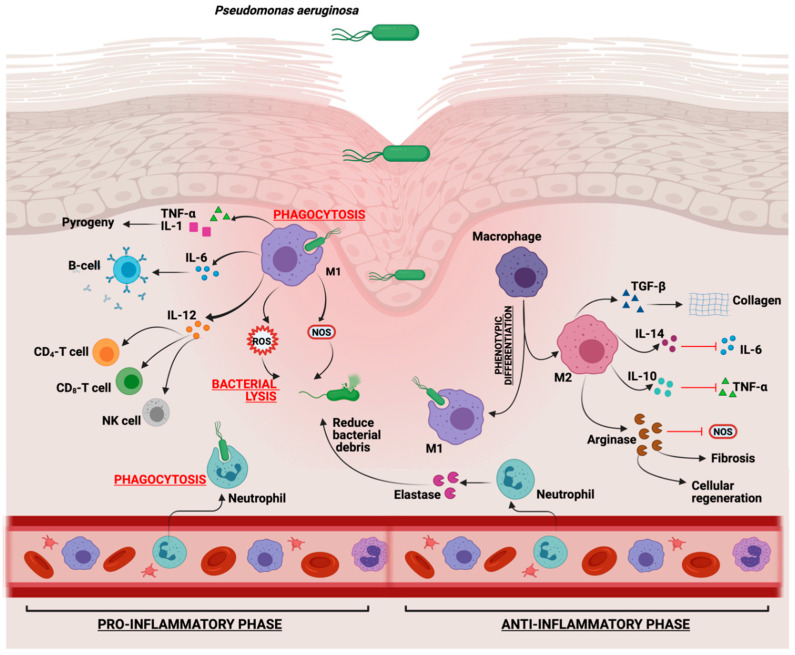
Normal immune response to *Pseudomonas aeruginosa*.

It is known that M1-macrophages act in the early inflammation phase and M2-macrophages interfere in the proliferation phase. Particularly, M1-macrophages produce pro-inflammatory cytokines such as tumor necrosis factor (TNFα), interleukin 6 (IL-6), interleukin IL-12, and antibacterial mediators such as reactive nitrogen and oxygen species (NO and ROS); M2-macrophages produce anti-inflammatory cytokines such as interleukin IL-4, interleukin IL-10, TGF, and arginase that promote tissue regeneration (Figure 2) [57].

*P. aeruginosa* in the wound healing environment promotes the pro-inflammatory genes expression in M1 macrophages because *P. aeruginosa* produces changes in the phenotype of macrophages and prolongs the M1-cells markers release, resulting in insufficient M2-macrophage signals, reduction in anti-inflammatory cytokines and growth factors TGFβ, and finally, the extension of the inflammation phase and delays healing [5,21].

## 7. Interaction of *Pseudomonas aeruginosa* with Other Pathogens on Skin Wound Healing Process

The skin is a physical barrier that offers protective niches and nutrients for different microorganisms facilitating their survival and at the same time generating competition and cooperation among them; but when this physical barrier is deteriorated, inflammatory conditions occur in the skin. Therapies focusing only on a primary pathogen are not usually successful due to the typical polymicrobial communities of the skin [64,65,66] where *P. aeruginosa* can interact with both pathogenic and commensal skin microorganisms. The common bacteria interacting with *P. aeruginosa* are *Staphylococcus aureus*, *Klebsiella pneumoniae* and *Streptococcus* spp., *Staphylococcus epidermidis*, and *Micrococcus luteus* (Table 3).

Under normal conditions, *P. aeruginosa* can suppress *S. aureus* growth by releasing a variety of toxins such as 4-hydroxy-2-heptylquinoline N-oxide (HQNO), pyocyanin, or LasA protease which inhibits the cytochrome system, the oxidative respiration reactions, and lyses *S. aureus* cells [30]. However, when the quorum sensing (QS) is deficient, a commensal-like interaction occurs between *S. aureus* and *P. aeruginosa*. It is also known that in these polymicrobial communities, one species always predominates. Using an antimicrobial method, Wilkinson y Hardman [20] found that *P. aeruginosa* becomes dominant when resources are limited, taking advantage of vital cofactors such as iron released by *S. aureus* when it is lysed by *P. aeruginosa*. They have also demonstrated that *P. aeruginosa* has a competitive advantage over *S. aureus* and *K. pneumoniae* by releasing toxic metabolites such as rhamnolipids or hydroxyquinolone. However, there is the possibility that at a certain point in the infection, when any strain of *P. aeruginosa* suffers an enrichment, a divergence of its population occurs and gives rise to the survival of pathogens such as *S. aureus* and *K. pneumoniae* (Table 3) [64].

*Pseudomonas aeruginosa* uses quorum-sensing molecules such as 3-oxo-C12-HSL and C4-HSL to regulate key virulence factors such as elastase, pyocyanin, rhamnolipids, and exotoxin A. These signals not only control their own pathogenicity but also inhibit the adhesion and biofilm formation of *Staphylococcus epidermidis*, giving *P. aeruginosa* a competitive advantage in polymicrobial wound environments [71]. This is achieved through the production of quorum-sensing molecules such as 2-heptyl-3-hydroxy-4-quinolone (PQS) and N-acyl homoserine lactones (AHLs), which interfere with the gene expression and metabolic activity of competing bacteria, thereby suppressing their colonization and enhancing the dominance of *P. aeruginosa* in chronic wounds [72].

Therefore, it can also help *P. aeruginosa* dominate under coinfection conditions. The *P. aeruginosa* extracellular polysaccharides (Pel and Ps1) have been shown to exert anti-staphylococcal activity by dispersing agents of *S. epidermidis* biofilms [73]. This occurs through a dual mechanism: Pel and Psl interfere with the structural integrity of the *S. epidermidis* biofilm matrix while also enhancing the penetration of antimicrobial compounds produced by *P. aeruginosa*, such as rhamnolipids and pyocyanin, which further inhibit staphylococcal viability and colonization in polymicrobial environments [40]. On the other hand, *P. aeruginosa* produces around 55 quinolones/quinolines molecules with significant antibiotic activity against Gram-positive bacteria [74]. Antimicrobial quinolines can be packaged into extracellular membrane vesicles (MVs) to cause direct *S. epidermidis* lysis, for example (Table 3) [73].

Interactions between *Pseudomonas aeruginosa* and *Streptococcus* species are frequently observed in cystic fibrosis-associated infections. Notably, *Streptococcus parasanguinis* has been shown to penetrate *P. aeruginosa* biofilms and disrupt their structure, thereby reducing the expression of key virulence genes. This occurs through the secretion of hydrogen peroxide (H_2_O_2_) and other diffusible inhibitory molecules that compromise the integrity of the *P. aeruginosa* exopolysaccharide matrix, leading to biofilm destabilization and downregulation of quorum-sensing systems such as *lasR* and *rhlR*, which are essential for the regulation of virulence factors including elastase and pyocyanin [75].

**Figure 3:**
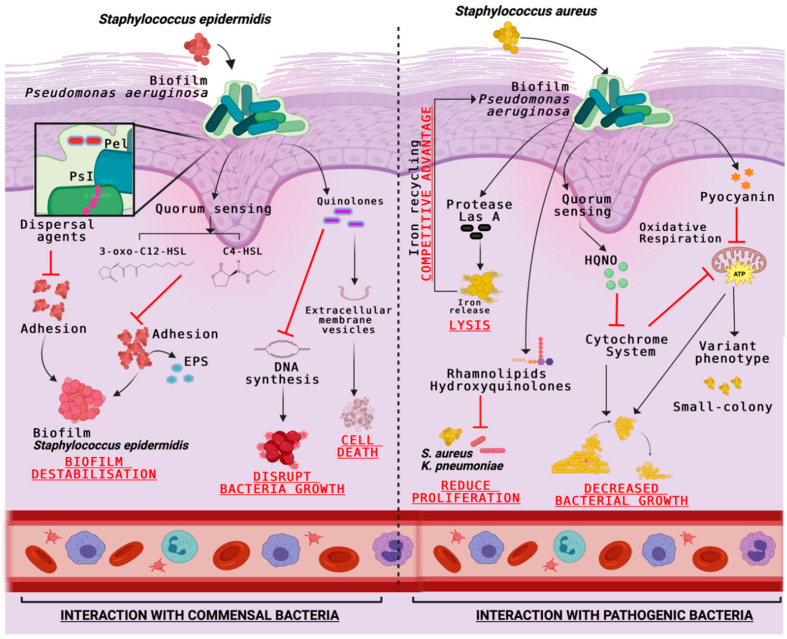
Interactions of *Pseudomonas aeruginosa* with commensal and pathogenic bacteria.

This interference impairs biofilm stability and limits the production of pathogenic factors, ultimately attenuating *P. aeruginosa* pathogenesis and enhancing the host’s ability to control infection [76]. *S. parasanguinis* is atypical on skin, however a similar interaction could occur between *P. aeruginosa* and another *Streptococcus* genus pathogens. In its interaction with Anginosus Group Streptococci, *P. aeruginosa* enhances the production of virulence factors such as pyocyanin and elastase, important molecules for biofilm formation. These virulence factors’ activity increase, causing considerable damage in the affected area of the patient (Figure 3) [77].

*P. aeruginosa* secretes acyl homoserine lactones (AHLs) with variable fatty acid side chains, which interfere with *Streptococcus pyogenes* hemolytic capacity. The mechanism includes the negative regulation of the *sag* operon expression, involved in the streptolysin S production, important for erythrocytes, leukocytes, and platelets lysing [70].

Moreover, the ability of *P. aeruginosa* to inhibit or promote the growth of other microorganisms significantly shapes the trajectory of wound healing. Inhibitory interactions, such as the production of HQNO, pyocyanin, or LasA protease against *Staphylococcus aureus*, may transiently suppress competing pathogens but often intensify local tissue damage, inflammation, and biofilm formation, ultimately delaying re-epithelialization [68,69,70]. Conversely, in certain polymicrobial contexts, cooperative interactions can increase overall virulence, leading to more severe infections and prolonged healing times [64,77].

## 8. *Pseudomonas aeruginosa* Skin Wound Infection: Current and Emerging Therapies

The best option to avoid damage by *P. aeruginosa* in epithelium lesions is to eliminate the bacterial cells. As a result, some treatments are using antibiotics such as levofloxacin, ciprofloxacin, gentamicin, amikacin or tobramycin, as the first line of treatment. Because *P. aeruginosa* has high resistance levels to most of the commonly used antibiotics, including aminoglycosides, quinolones, and β-lactams, and also to the common disinfectants, this makes it difficult to definitively eradicate from the infected tissue [78,79]. Furthermore, antibiotics can target only vegetative cells but poorly penetrate the biofilm matrix [80]. At present, aminoglycoside antibiotics and azithromycin are widely used to treat *P. aeruginosa* infections [81]. However, the treatment effectiveness depends on the correct dose to eliminate all bacterial cells and avoid side effects. Antibiotics synergy has been considered as a good alternative because it requires a lower dose of each antibiotic. For example, combining azithromycin and gentamicin with ceftolozane and tazobactam have had significant results in the eradication of *P. aeruginosa* [82,83].

The relentless rise in multidrug-resistant *Pseudomonas aeruginosa* underscores the need for adjuvant or alternative therapies that do not rely solely on conventional antibiotics. Several antimicrobial peptides, cold atmospheric plasma, non-classical modalities—photodynamic therapy, mesenchymal stromal cells (MSCs) and their extracellular vesicles (EVs), as well as mitochondrial transplantation [84,85] have emerged as promising tools to disrupt biofilms and accelerate wound closure.

Antimicrobial peptides are promising therapeutic options to treat *P. aeruginosa* infections that reduce the negative impact of virulence factors on host cells by preventing biofilm formation and also favors skin epithelial repair by inducing the migration of keratinocytes to the affected area that promote wound healing [84]. *P. aeruginosa* facilitates this process through the secretion of specific bioactive molecules, such as lipoxygenase-derived oxylipins, which modulate host cell signaling pathways, including MAPK and PI3K/Akt, thereby stimulating keratinocyte migration and proliferation essential for re-epithelialization [86].

Cold plasma used in skin injury therapy inhibits microbes in chronic wounds due to its antiseptic effects, since it has been used as a sterilizing agent. Its efficacy arises from the generation of a mixture of reactive oxygen and nitrogen species (RONS), UV photons, and transient electric fields, which together disrupt microbial cell walls, damage nucleic acids, and inactivate essential enzymes. Unlike conventional antiseptics, cold plasma acts non-thermally and can penetrate biofilms without harming surrounding healthy tissue, making it particularly effective against multidrug-resistant bacteria commonly found in chronic wounds. Moreover, it has been shown to promote wound healing by enhancing local microcirculation and stimulating keratinocyte and fibroblast proliferation [87].

In addition, it is a source of gaseous nitric oxide which works as a powerful microbicide agent that destroys different kinds of bacteria, while it promotes healing by proliferation cell stimulation [88]. The aim of the topical therapy is to reduce bacteria count in the wound and remove soluble debris without adversely affecting cellular activities during the wound healing process.

Furthermore, photodynamic therapy (PDT) has shown considerable potential in inhibiting *Pseudomonas aeruginosa* biofilm formation and accelerating the wound healing process. PDT functions through the administration of a photosensitizing agent that selectively localizes within bacterial cells. Upon exposure to a specific wavelength of light, this agent becomes activated and produces reactive oxygen species (ROS), such as singlet oxygen and free radicals. These ROS induce oxidative stress that damages bacterial membranes, proteins, and DNA, ultimately leading to cell death. In the case of *P. aeruginosa*, oxidative damage disrupts the biofilm matrix and inhibits its growth, allowing the host immune system and tissue repair mechanisms to function more effectively [89].

New methods are currently being studied to improve tissue repair, such as the use of mitochondrial transfer due to its potential to revitalize senescent cells. The mitochondria could stop the production of free radicals, thus reducing the recovery timing from a wound and possible complications [90]. Mitochondria are the main source of reactive oxygen species (ROS) that are involved in all stages of the tissue repair process. At low concentrations, they are essential participants in cell signaling, induction of the myogenic response, and defense against infectious agents [90]. In pre-clinical models, MSC-EVs enhance standard antibiotic regimens by (i) delivering microbicidal molecules that curb bacterial replication, (ii) re-programming macrophages toward a pro-resolution (M2) phenotype, and (iii) releasing pro-angiogenic and anti-apoptotic factors that stimulate keratinocyte migration and tissue regeneration [91,92,93,94]. For example, in a rabbit model of renal tuberculosis, MSC-EVs potentiated first-line anti-tubercular drugs, tempered inflammation, and minimized parenchymal damage [93]. NADPH leukocyte oxidase (Nox) is one of the main sources of ROS involved in pathogen destruction as well as vascular endothelial growth factor (VEGF) signaling, and TNF response [90]. However, when concentration exceeds the balance between production and uptake, a phenomenon known as oxidative stress is generated, which is a key factor that delays healing because it significantly alters the microbiome by promoting the colonization of biofilm-forming bacteria [95,96].

In addition to pharmacological approaches, wound bed preparation through debridement is a cornerstone in chronic wound management. Debridement physically removes necrotic tissue, bacterial biofilms, and cellular debris, reducing the bioburden and facilitating the penetration of topical or systemic antimicrobial agents [91]. Various methods, such as surgical, enzymatic, autolytic, and mechanical debridement, have been shown to enhance granulation tissue formation and create a favorable microenvironment for subsequent healing phases [91].

Equally important is promoting epithelial repair, which involves stimulating keratinocyte migration and proliferation to restore the skin barrier. Strategies include the application of bioactive dressings enriched with growth factors, extracellular matrix components, or antimicrobial peptides, which not only inhibit *P. aeruginosa* colonization but also accelerate re-epithelialization [91,92,93]. By integrating these regenerative interventions alongside infection control, wound healing outcomes can be significantly improved.

Further approaches have been revised like the restoration of microbial balance. Strategies include the use of probiotics, bacteriophage therapy, and topical antimicrobials with selective activity that modulate the microbiota without completely eradicating beneficial species [75]. Additionally, quorum-sensing inhibitors and biofilm-disrupting agents can reduce *P. aeruginosa* dominance, allowing commensal species to re-establish and support wound repair [77]. These approaches aim to shift the microbial community toward a non-pathogenic state, thereby reducing inflammation, enhancing granulation tissue formation, and accelerating closure of chronic wounds.

## 9. Conclusions

*Pseudomonas aeruginosa* plays a critical pathogenic role in skin wound healing by interfering with multiple phases of the repair process, especially inflammation and proliferation. Through the secretion of exotoxins and the formation of structurally complex biofilms composed of alginate, Pel, Psl, and extracellular DNA, the pathogen delays wound closure and promotes chronic infection.

The virulence of *P. aeruginosa* is regulated by quorum-sensing systems (las and rhl) and intracellular signaling molecules such as cyclic di-GMP, which coordinate the expression of key factors including pyocyanin, exotoxin A, elastases, and rhamnolipids. These molecules disrupt host immune responses, degrade extracellular matrix components, and prevent effective epithelial regeneration.

The capacity of the pathogen to evade immune defenses is reinforced by its interaction with host proteins such as vitronectin, suppression of complement activation, and modulation of macrophage phenotypes, prolonging the pro-inflammatory M1 phase and delaying the anti-inflammatory M2 transition required for tissue regeneration.

The *P. aeruginosa* maintains a competitive advantage in polymicrobial environments by inhibiting adhesion and biofilm development of other pathogens like *Staphylococcus epidermidis* and *Streptococcus pyogenes* through the release of AHL signals, quinolones, and other antimicrobial compounds. However, certain streptococcal species, such as *Streptococcus parasanguinis*, can disrupt *P. aeruginosa* biofilms and reduce its virulence.

Therapies such as antibiotics, peptides, and other novel alternatives such as mitochondrial application help to control the pathogenic potential of *P. aeruginosa* in the wound healing environment.

## Figures and Tables

**Table 2:** Cells and molecules interacting between *Pseudomonas aeruginosa* and the immune system.

Cells	Secreted Molecules		Description	Reference
Immune System				
M1 macrophages	Pro-inflammatory cytokines	Tumor Necrosis Factor (TNFα)	Cytokine released immediately after any damage by exposure to bacterial LPS. Acts through two transmembrane receptors: TNF receptor 1 (TNFR1) induces programmed cell death and TNF receptor 2 (TNFR2) is responsible for cell proliferation. Therefore, depending on the cell type, TNFR1 and TNFR2 may have distinct roles in signal transduction and gene expression.	[58]
		IL-6	Interleukin that promotes T and B lymphocytes differentiation and maturation, stimulates immunoglobulin release by B cells and pro-inflammatory cytokines inhibition such as TNF-α that participates in the macrophage M1 to M2 maturation.	[59]
		IL-12	Interleukin that activates T CD_4_^+^ (H1) type 1 cells and stimulates the NK cells and T CD_8_^+^ lymphocytes production.	[60]
	ROS/NOS	Reactive oxygen and nitrogen species that cause significant cell structures damage causing cell lysis.		[61]
M2 macrophages	Anti-inflammatory cytokines	TGF-β	Interleukin that protects the collagen expression by some protease activity inhibition.	[62]
		IL-14	Interleukin that inhibits the pro-inflammatory cytokines synthesis such as TNF-alpha and IL-6.	[60]
		IL-10	Interleukin that inhibits the pro-inflammatory cytokines synthesis such as IFN-γ, IL-2, IL-3, and TNFα.	[60]
	Arginase	Enzyme responsible for NOS synthesis regulation and tissue regeneration.		[62]
Neutrophils	Elastases	Protease that is released as a defense mechanism to remove NOS and ROS tissue degradation products.		[63]
*Pseudomonas aeruginosa*	Exotoxin A	Modifies macrophage gene expression and inhibits the maturation of M1 macrophages to M2.		[56]
	Alginate	Inhibits bacterial uptake during phagocytosis in Macrophages and Neutrophils.		[34]
	Exotoxin A	Modify gene expression to cause apoptosis in Neutrophils.		[56]

**Table 3:** Molecular and cellular interactions during wound healing process in the *P. aeruginosa* presence.

Pseudomonas aeruginosa Secreted Molecules	Cells Interaction with	Description	Reference
Quinolones	*Staphylococcus epidermidis*	Molecule packaged in extracellular membrane vesicles (MVs) to cause cell lysis.	[67]
Pel and PsI		Both acts as dispersing agents to inhibit biofilm formation and adhesion.	[67]
3-Oxo-C12-HSL		Inhibits the bacterial growth and EPS secretion that hinders the initial adhesion and the biofilms formation.	[67]
4-hydroxy-2-heptylquinoline N-oxide (HQNO)	*Staphylococcus aureus*	Quinolone signal system component acts as an inhibitor of *S. aureus* electron transport chain (ETC). Prolonged exposure to this compound leads to the small colony variants selection.	[68]
Pyocyanin		Increases the H_2_O_2_ formation and leads to cell lysis.	[68]
Las A		Protease that lyses *S. aureus* cells.	[68]
Pyocyanin	*Streptococcus* spp.	Increases the H_2_O_2_ formation and leads to cell lysis.	[69]
Acyl homoserine lactone (AHL)	*Streptococcus pyogenes*	Modifies hemolytic activity and reduces pathogenicity of *S. pyogenes*.	[70]

## Data Availability

No new data were created or analyzed in this study. Data sharing is not applicable to this article.
